# Internal Herniation Incidence After RYGB and the Predictive Ability of a CT Scan as a Diagnostic Tool

**DOI:** 10.1007/s11695-020-04892-8

**Published:** 2020-08-03

**Authors:** Bart Torensma, Laurens Kooiman, Ronald Liem, Valerie M. Monpellier, Dingeman J. Swank, Larissa Tseng

**Affiliations:** 1 Department of Anaesthesiology, Leiden University Medical Center, Leiden, The Netherlands; 2 Department of Surgery, Dutch Obesity Clinic West & LUMC, The Hague, The Netherlands

**Keywords:** Bariatric surgery, Morbid obesity, RYGB, Internal herniation, CT scan, Incidence, Diagnostic tool

## Abstract

**Purpose:**

The clinical diagnosis of an internal herniation (IH) after a Roux-en-Y Gastric Bypass (RYGB) remains difficult; therefore, performing a CT scan is usually part of the diagnostic process. The goal of this study was to assess the incidence of IH in patients with open and closed MD (mesenteric defect) and to study if the ability to diagnose an IH with a CT scan is different between these groups.

**Materials and Methods:**

IH was defined as a visible intestine through the mesenteric defect underneath the jejunojejunostomy and/or in the Petersen’s space. CT scan outcomes were compared with the clinical diagnosis of an IH. Until 31 June 2013, standard care was to leave mesenteric defects (MDs) open; after this date, they were always closed.

**Results:**

The incidence of IH in the primarily non-closed group was 3.9%, and in the primarily closed group, this was 1.3% (*p* = 0.001). In group A (non-closed MD and CT), the sensitivity of the CT scan was 80%, and specificity was 0%. In group C (closed MD and CT), the sensitivity was 64.7%, and specificity was 89.5%. In group B (non-closed, no CT), an IH was visible in 58.7% of the cases and not in 41.3%. In group D (only a re-laparoscopy), an IH was visible in 34.3% of the cases and not in 65.7%.

**Conclusions:**

Using the CT scan in suspected IH is not useful in if the MDs were not closed. If the MDs were closed, then a CT scan is predictive for the diagnosis IH.

## Purpose

The internal herniation (IH) is a well-known late complication of the Roux-en-Y gastric bypass (RYGB). Since the introduction of this complication in a case report in 1999 by Serra et al. [1], numerous publications have been dedicated to IH incidence, prevention, and diagnostics. Despite these publications, the clinical diagnosis of an IH remains challenging. Moreover, there is still no consensus on standardized closing of mesenteric defects (MD) and the use of a computer tomography (CT) scan for the diagnosis of the IH [2–10].

In daily practice, the CT scan is widely used as a diagnostic tool for IH. Most previous trials validating the CT scan for detecting IH show a large variety in sensitivity and specificity, and have low power due to too few inclusions or algorithm for detecting IH from a CT scan [9, 11–17]. Thus, the value of the CT scan in IH diagnostics is unclear. Furthermore, it is also uncertain whether the sensitivity and specificity of the CT scan are different when mesenteric defects were closed or left open.

Only three studies with a large population and assumable enough power have shown results towards a better understanding of the effect of the CT scan on detecting IH. Since 2018, two large trials in 1475 bariatric patients showed that the CT scan had a specificity of 87.1% and a high negative predictive factor of 96.8% [18, 19]. Also, a study [20] found in 40.2% of the time an IH on the CT scan, and at the same time, the study could in 61.1% objectify this finding during surgery.

However, up to date, there is still discussion as to whether closing the MD leads to a lower incidence of IH and what the role of a CT scan is in the diagnostic algorithm. Our clinic changed its policy in 2013 from leaving MD open to standardly closing the mesenteric defects. This gives us the opportunity to study the incidence of IH in patients with and without closed MD. In addition, we can assess whether the accuracy of a CT scan is different in patients with and without closed MD.

## Materials and Methods

### Study Population

This is a retrospective single-center cohort study. All patients who underwent a primary laparoscopic RYGB between January 2011 and December 2016 were selected from hospital electronic databases. All patients gave informed consent at the start of the treatment. All data were used anonymously.

### Standard Treatment

All patients were screened and indicated according to the IFSO criteria prior to bariatric surgery. Patients received pre- and postoperative multidisciplinary counseling focused on long-term behavioral change [21]. This program has a 5-year follow-up.

Regarding the closure of the MD, standard policy changed during study period. Between January 2011 and June 2013, the MD were never closed when a RYGB was performed: the *non-closed group*. From July 2013 until December 2016, all mesenteric defects where closed with the stapler device EMS from Johnson & Johnson: the *closed group*. Halfway this second period, the clinic changed towards the hernia stapler Universal by Medtronic. The same technique was used, and, in both devices, there were no differences in the height of the staples. Patients were operated by four surgeons; all have performed more than 2000 RYGBs and sleeves since 2011 until now (2019).

### Patient Selection

Patients were retrospectively selected from the electronic hospital database to see if they had undergone a re-laparoscopy after RYGB or not.

A patient was selected if it was announced at the emergency department with complaints with intermitted pain in the upper (left) abdomen, related to complaints after a meal with or without other obstruction complaints. If so, they got a re-operation through a re-laparoscopy. A CT scan was done in cases whereby the complaints could predict alternative diagnoses.

A CT scan was done as check and always diagnosed by a radiologist who reported this in the patient electronic file. Re-operation was done in 100% of the cases. For analysis, patients were divided in four groups:

- Group A is the non-closed group who underwent a CT
- Group B is the non-closed group without CT.
- Group C is the closed group who underwent a CT.
- Group D is the closed group without CT scan.

### Internal Herniation

An IH was diagnosed during re-operation if they met the following criteria:

1. Internal hernia, defined as the presence of herniated small bowel with or without obstruction or ischemia through visible opening in the MD underneath the jejunojejunostomy and/or the Petersen’s space. Incidental IHs, found at laparoscopies conducted for uncertain abdominal pain, were included as having IH.
2. Suspected intermittent IH defined as clinical suspicion of IH and/or signs on CT scan, but at laparoscopy presenting open mesenteric defects without intestinal loop.
3. For the incidence calculation, the findings of the surgeon during the operation were used and compared with the findings of the radiologist report.

### Patient Characteristics

Obtained data included age, sex, preoperative weight, postoperative weight, IH presence, IH in date, and time period. Body mass index (BMI) and percentage total weight loss (%TWL) were calculated.

### Individual Differences Calculation Between Surgeons

Since an operation is a human action, variation of closing technique could predict the incidence of an IH. Therefore, the incidence of IH was tested and calculated for each of the four surgeons.

### Data Capture

The analysis was performed on a blinded data set after a medical/scientific review was completed, and all protocol violations have been identified and the data set was declared complete. All data were collected in a data management system (Castor EDC, Amsterdam, The Netherlands; https://www.castoredc.com) and handled according to Good Clinical Practice guidelines, Data Protection Directive certificate and complies with Title 21 CFR Part 11. Furthermore, the datacenter where all the research data is stored is ISO27001- and ISO9001-certified and Dutch NEN7510-certified.

### Statistics

For analyses, descriptive statistics and inferential statistics were used. All data was first tested for normality by a Kolmogorov-Smirnov test, a Q-Q plot, and Levene’s test.

Categorical variables were expressed as *n* (%). Continuous normally distributed variables were expressed by their mean and standard deviation, not normally distributed data by their median and min-max range for skewed distributions. To test groups, categorical variables were tested using the Pearson chi-square test or Fisher exact test, when appropriate. Normally distributed continuously unpaired data were tested with the independent samples Student’s *t* test and, in case of skewed data, with the independent samples Mann-Whitney *U* test.

Normally distributed continuously paired data were tested with the dependent samples Student *t* test and, in case of skewed data, with the Wilcoxon signed-rank test.

Intraclass correlations and sensitivity/ specificity were calculated on specific data in the set. Moreover, an intraclass correlation test was performed to check the difference between surgeons, with regard to incidence of IH. Significance level was set at *p* value < 0.05.

Statistical analysis was performed using R studio statistical software (version 1.0.153).

### Sensitivity and Specificity

In this study, statistics about sensitivity and specificity is described as follows:

Sensitivity: With a high sensitivity, you can assume that a patient is really not ill with a negative test result and can therefore go home. When the sensitivity decreases, the number of false negatives will increase.

Specificity: With a high specificity, you can assume that a patient is really ill with a positive test result and you can start treatment. When the specificity decreases, the number of false positives will increase.

In this manuscript, the focus will be on “yes” or “no” starting a treatment and therefore the specificity of the results.

## Results

### Baseline Demographics

Between the period of January 2011 and December 2016, 3262 patients underwent a RYGB. The database study found a total of 133 patients (4.1%) who were re-operated for suspected IH and were included in this study. Most of these patients were female 119 (89.5%). Mean ± SD age was 44.56 ± 9.7 years old. The mean ± SD preoperative BMI before bariatric surgery was 43.3 ± 12.1 kg/m^2^. At the day of re-operation, the mean ± SD BMI was 29.7 ± 6.5 kg/m^2^ and TWL was 31.1%. The average time between RYGB and IH was 17.98 ± 11.2 months.

There were no significant differences in baseline characteristics between the patients who were diagnosed or suspected with IH and the patients who were not.

In 56 patients (42.1%), the re-operation was performed in an acute phase, and in 77 patients (57.9%), elective surgery was scheduled (*p* = 0.351). In nine cases (6.8%), laparoscopy was converted because of insufficient exposure. All other cases (*n* = 124/93.2%) were successfully performed laparoscopically (*p* = 0.03).

### Closed Versus Open Mesenteric Defects

**Fig. 1:**
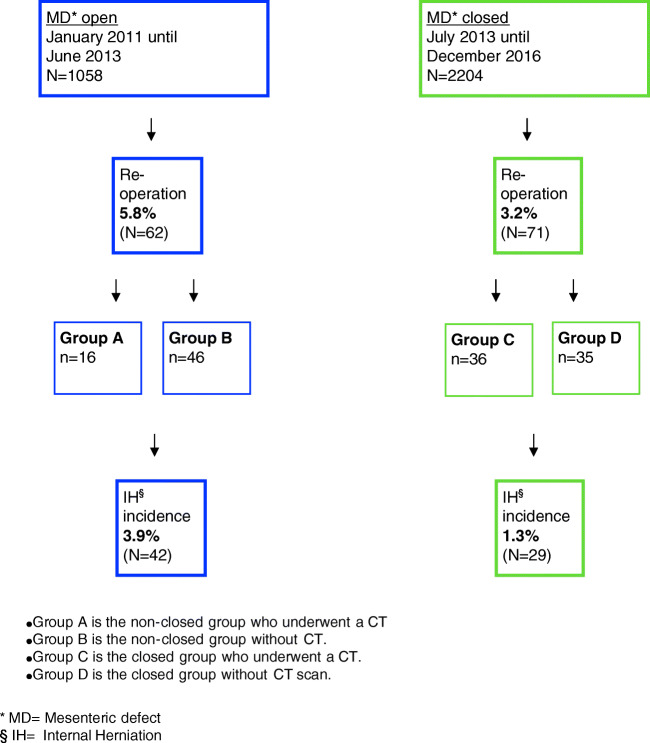
Group A is the non-closed group who underwent a CT. Group B is the non-closed group without CT. Group C is the closed group who underwent a CT. Group D is the closed group without CT scan. MD, mesenteric defects; IH, internal herniation

Between January 2011 and June 2013 (MD left open), 1058 patients underwent a RYGB; 62 of these patients were suspected of IH and underwent a re-operation. In the group of patients with closed MD (*N* = 2204), 71 patients underwent a re-operation. Thus, the incidence of re-operation in the *non-closed group* was significantly higher (5.8%) than that in the *closed group* (3.2%, *p* = 0.001). The incidence of IH was also significantly higher in *non-closed group* (3.9%) compared with that in the *closed group* (1.3%, *p* = 0.001, Fig. 1).

### Computer Tomography Scan and Diagnosis of Internal Herniation

Group A, *non-closed group* who underwent a CT scan, consisted of 16 patients. In this group, the sensitivity of the CT scan was 80% and specificity was 0%. The positive predictive value of the CT scan in this group was 92%, and the negative predictive value was 0%.

Thus, with the high *sensitivity*, you can assume that a patient is really not sick with a negative test result and no treatment is possible and the patient can go home. When the sensitivity decreases, the number of false negatives will increase. The risk is that people who are sick will be sent home. Whereby, the positive predictive value indicates the after-chance of the presence of disease in people with a positive test result. Since we are interested in the fact of starting a treatment yes or no, statistical wise, we need to look at the *specificity* results.

Group B was the *non-closed* group who was re-operated without a performance of a CT scan (*N* = 46). In this group, an IH was visible during re-operation in 58.7% of the cases, and in 41.3%, it was not.

Group C was the *closed group* in whom a CT scan was performed (*N* = 36). In group C, the sensitivity of the CT scan was 64.7% and specificity of 89.5%, and a positive predictive value of 84% and a negative predictive value of 74%. Thus, with a high *specificity*, you can assume that a patient is really ill with a positive test result and you can start the treatment.

Group D was the *closed group* without CT scan (*N* = 35). In group D, where only a re-operation was done, an IH was visible in 34.3% of the patients, and in 65.7%, it was not.

### Follow-up

In group A, 81.3% of patients were symptom-free after re-operation; this was 76.1% in group B, 61.1% in group C, and 60.0% in group D. Thus, a total of 42 patients had IH surgery but still suffered from abdominal pains afterwards.

### Intraclass Correlation Between Surgeons and IH Occurring

A total of 27.1% of the IH occurred in patients who were operated by surgeon A; in patients who were operated by surgeons B, C, and D, a total of 24.3% of the patients developed an IH. The intraclass correlation was 98% (*p* = > 0.05). Thus, there was no difference in incidence of IH occurring between the four surgeons and their surgical technique.

## Discussion

The goal of this study was to assess the incidence of IH in patients with open and closed MD and to study if the ability to diagnose an IH with a CT scan was differed between these groups. In the Netherlands, the policy within bariatric surgery is that every patient will go back towards their treating hospital. Lost to follow, when complaints occur up on internal herniation, is therefore not possible in our database. Every surgeon saw their own patient postoperatively.

The results showed that the incidence of IH was significantly lower in patients with closed MD. Our data show that the incidence of IH can be significantly reduced by closing the MD: the incidence dropped from 3.9 to 1.3%. These results are comparable with other studies [2, 22–27].

This study had a quite low incidence of IH in patients with open MD. It was 3.9% compared with 11.7% in the study by Aghajani et al., which is considered the golden standard [27]. Both studies had enough power (*N* = 3226 versus 4013) and over 5 years follow-up (2005/2015 compared with this study 2011/2017). Therefore, we cannot explain the differences.

Previous research focused on the CT scan in IH diagnostics [10–17]. However, none of these studies assessed the difference in CT accuracy comparing patients with open and closed MD in a clinical setting. Our results showed that the diagnostic accuracy of a CT scan in supporting the choice of treatment is not useful in patients with an open MD. In this group the CT scan had a specificity of 0%; this means the risk is very high that people are operated while they do not have an IH. The possible explanation for the low predictive value of the CT scan in this group may be that the intestines move more freely (i.e., higher change for intermittent IH). So, it could be that during the CT scan, there was an IH, but when performing the laparoscopy, the intestines were returned to their normal anatomic situation.

In the *closed group*, the CT scan had a very high specificity (89%). When patients in the *closed group* were directly operated without performing a CT scan, there was a large group (65.7%) who did not have an IH and the re-operation performed was “unnecessary.” Considering these results, in combination with the high specificity of the CT scan in this group, we recommend performing a CT scan when a patient with closed MD is suspected of IH.

It is important to appreciate that a CT scan can never rule out an IH completely. Thus, if symptoms of an IH remain, a laparoscopy is inevitable to exclude an IH as cause of the complaints.

After the correction of the IH and closure of the MD, it was striking that three out of ten patients still had abdominal complaints. This suggests that, in addition to the IH, there are other, possible unknown causes for abdominal pain after RYGB. A study with the results of 5 years postoperative after bariatric surgery showed that chronic abdominal pain was reported by 33.8%. Also, symptoms of indigestion and irritable bowel syndrome were reported by 48.8% and 29.1% [28]. Multi-center/country prospective long-term follow-up studies are necessary to understand the causes of these abdominal complaints.

Especially since the ability of scoring pain and the perception of pain is different in patients with obesity compared with patients without obesity [29]. This may have a significant effect on pain treatment and consequently needs to be taken into account when treating the patients with obesity for acute or chronic pain [29, 30].

A limitation of this study is the fact that this is a retrospective cohort; we have no knowledge of the current abdominal complaints of the patients who were not free of symptoms after re-operation. The fact that a CT scan was not performed in all patients could be perceived a limitation. Moreover, the results give us new insights on operating for suspected IH when mesenteric defects are primarily closed or open.

## Conclusion

The incidence of IH can be significantly reduced by closing the mesenteric defects. The diagnostic accuracy of a CT scan in supporting the choice of treatment is not useful in a patient with open MD. However, when a patient with closed MD is suspected of IH, a CT scan can be predictive for the diagnosis of an IH.

After the correction of an internal herniation, three out of ten patients still have postoperative complaints. This suggests that, in addition to an IH, there are other causes for abdominal pain after RYGB.
